# Molar Heat Capacity (C_v_) for Saturated and Compressed Liquid and Vapor Nitrogen from 65 to 300 K at Pressures to 35 MPa

**DOI:** 10.6028/jres.096.047

**Published:** 1991

**Authors:** J. W. Magee

**Affiliations:** National Institute of Standards and Technology, Boulder, CO 80303

**Keywords:** calorimeter, heat capacity, high pressure, isochoric, liquid, measurement, nitrogen, saturation, vapor

## Abstract

Molar heat capacities at constant volume (*C_v_*,) for nitrogen have been measured with an automated adiabatic calorimeter. The temperatures ranged from 65 to 300 K, while pressures were as high as 35 MPa. Calorimetric data were obtained for a total of 276 state conditions on 14 isochores. Extensive results which were obtained in the saturated liquid region (*C_v_*^(2)^ and *C_σ_*) demonstrate the internal consistency of the *C_v_* (*ρ,T*) data and also show satisfactory agreement with published heat capacity data. The overall uncertainty of the *C_v_* values ranges from 2% in the vapor to 0.5% in the liquid.

## 1. Introduction

Accurate measurements of thermodynamic properties, including heat capacity, are needed to establish behavior of higher order temperature derivatives of an equation of state *P*(*ρ*, *T*). In particular, the heat capacity at constant volume (*C_v_*) is related *to P*(*ρ,T*) by:

$$C_{v}-C_{v}^{0}=-T\int_{0}^{\rho }{\left(\partial^{2}P/\partial T^{2}\right)}_{\rho }d\rho /\rho^{2}$$ (1)

where $C_{v}^{0}$ is the ideal gas heat capacity. Consequently, *C_v_* measurements which cover a wide range of (*P*, *ρ*, *T*) states are beneficial to the development of an accurate equation of state for the substance.

The amount of experimental data on the calorimetric properties of nitrogen is very limited. Measurements of *C_v_* are mostly confined to atmospheric pressure. Only two published works concern *C_v_* measurements at elevated pressure. First, Voronel et al. [1] obtained 69 experimental values of *C_v_* at temperatures from 106 to 167 K at densities close to the critical density, with an emphasis on the temperature variation of *C_v_* near the critical point (126.2 K). However, these authors give neither the pressures nor the densities at which the measurements were performed. Thus, comparisons with the *C_v_* data of Voronel et al. are difficult at best. In the only other experimental study at elevated pressures, Weber [2] performed measurements at temperatures from 91 to 242 K using the same calorimeter as used in this work. The combined works of Voronel et al. and Weber leave gaps in the *C_v_* surface between the triple point (63.15 K) and 91 K, and for temperatures above 242 K.

Published experimental results for the heat capacity of the saturated liquid (*C_σ_*) are more available than those for *C_v_* at elevated pressures. When combined, the data of Weber [2], Giauque and Clayton [3], and Wiebe and Brevoort [4] range from 65 to 125 K. However, none of these works spans the entire temperature domain for saturated liquid nitrogen. Thus, we cannot be certain how the various data will intercompare. In this work, the goals include extending the published data for *C_v_* to temperatures as low as the triple point, and also to near ambient temperature. A second goal is to measure *C_σ_* over temperatures from the vicinity of the triple point to that of the critical point, and to compare these measurements with published data and with predictions from published equations of state.

## 2. Experimental

**Figure 1:**
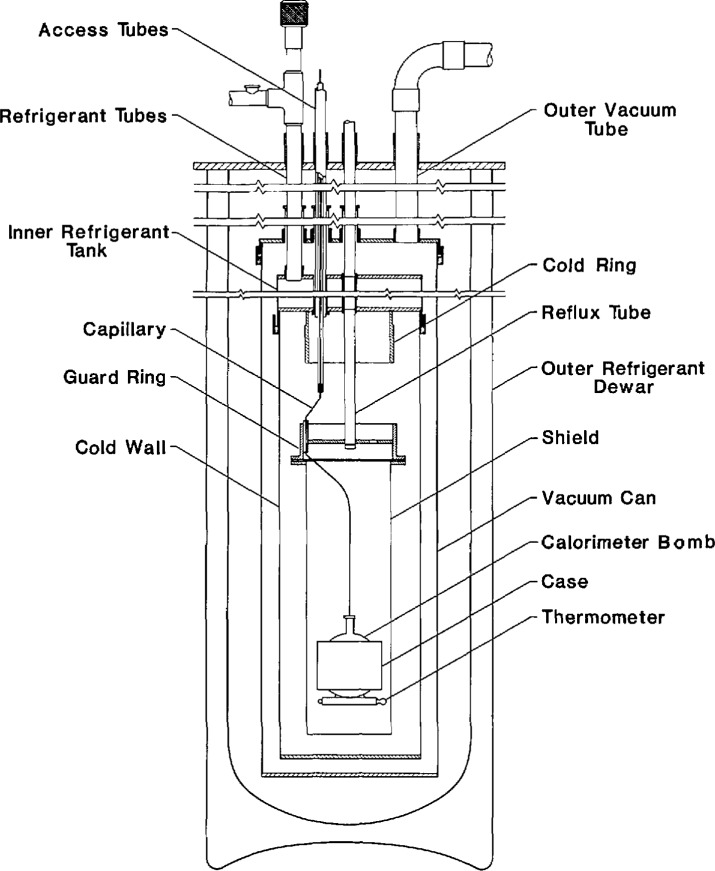
Details of the adiabatic calorimeter.

**Figure 2:**
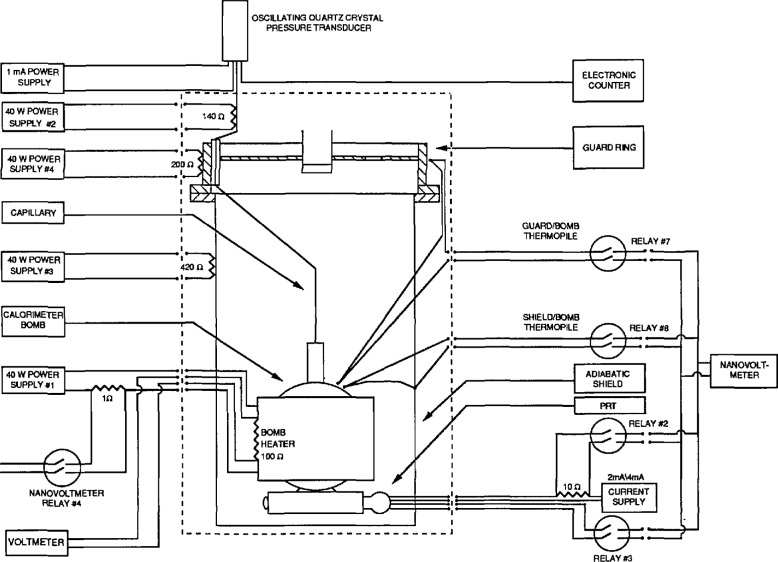
Instrumentation of the adiabatic calorimeter.

The apparatus in Fig. 1 has a long history dating to its original construction for liquid hydrogen calorimetry. Its mechanical details remain essentially unchanged since they were described by Goodwin [5]. Instrumentation, however, has been changed extensively. The older instruments were replaced with electronic versions, each equipped with an IEEE-488 standard interface for two-way communication with a microcomputer. The arrangement of the new instruments is shown in Fig. 2.

An experimental heat capacity is the applied heat (*Q*) corrected for the heat applied to the empty calorimeter (*Q*_0_) per unit temperature rise (Δ*T*) per mole (*N*) of substance. In terms of the observed measurements, the heat capacity is given by,

$$C_{v}=(Q-Q_{0})/(N\Delta T).$$ (2)

The applied heat is the product of the time-averaged power and the elapsed time of heating. Measured power is the product of instantaneous current and potential applied to the 100 Ω heater wound on the surface of the calorimeter bomb. During a heat measurement a series of five power measurements with an accuracy of 0.01% were made at 100 s intervals. Time was determined with a microcomputer clock to a resolution of 10^−4^ s. Elapsed time was computed with an accuracy of 0.001%. The heat (*Q*_0_) applied to the empty calorimeter has been determined by several series of calorimetric experiments on a thoroughly cleaned and evacuated sphere. These results include those of Roder [6] from 86 to 322 K and of Mayrath [7] from 91 to 340 K in addition to new data from 29 to 99 K, as presented in Table 1. The combined *Q*_0_ data sets were fitted to the expression,

$$Q_{0}(T)=\sum_{i=1}^{12}C_{i}(T_{2}^{i}-T_{1}^{i})$$ (3)

by applying a chord-fitting method to Δ*T* values ranging from 0.5 to 20 K. Details will follow in a later section.

Temperatures were measured with an automated circuit consisting of a 25 Ω encapsulated platinum resistance thermometer calibrated on the IPTS-68 by the NIST Temperature and Pressure Division, a 10 Ω standard resistor calibrated by the NIST Electricity Division, a stable (±2 ppm) electronic current source, and a bank of ultralow thermal emf (<1×10^−10^ V/K) relays multiplexing a precise nanovoltmeter. Potential measurements were made with the thermometer current flowing in both forward and reverse directions. An average thermometer resistance was calculated in order to avoid errors from spurious emfs. It is thought that the absolute temperatures derived this way are accurate within 0.03 K and precise to ±0.002 K. During this work we reproduced the generally accepted triple point temperature of N_2_ to less than 0.002 K as a further check on the validity of this claim. Temperature rises (Δ*T*) were established within 0.004 K by linear extrapolation of the preheating and the post-heating temperature drift data to the midpoint time of the heat cycle.

Pressure was measured with an oscillating quartz crystal pressure transducer whose signal was fed to a precise timer/counter. This instrument had a range of 70 MPa and was calibrated with a piston gauge. The experimental uncertainty of the measured pressure is estimated to be ±0.01% of full scale at pressures above 3 MPa, or ±(0.03–0.05)% of the pressure at lower pressures. Finally, the number (*N*) of moles of substance in the calorimeter is the product of the calorimeter volume (*V*_bomb_) and the molar density (*ρ*) derived from the equation of state [8] which has an uncertainty of ±0.1%. The calorimeter volume was obtained from a previous calibration [9] as a function of temperature and pressure, and is accurate to ±0.1%. The value of *N* derived in this way is believed to have an uncertainty of ± 0.2%. If a weighing method was used to evaluate *N*, the error would drop to ±0.01%. Other details will follow in a later section.

The spherical bomb depicted in Fig. 1 is constructed of Type 316 stainless steel with a wall thickness of 0.15 cm and an internal volume of 72.739 cm^3^ at 100 K. To prepare for an isochoric experiment, N_2_ was charged at a pressure of 10 MPa and at a suitable bomb temperature until the target density was obtained. Then the sample was cooled to near 63 K with liquid Ne refrigerant, or to near 80 K when liquid N_2_ was used. Each run commenced in the vapor + liquid region. The heater power was set to obtain about a 4 K temperature rise during each experiment. Apparatus control was then turned over to the microcomputer. A Fortran program was responsible for control of the cell heater. The guard and shield heaters followed the rise of the cell temperature using a specially tuned proportional-integral-derivative algorithm [10]. The program recorded, at periodic intervals, the bomb temperature, the cell pressure, and the voltage and current applied to the cell heater. Another Fortran program calculated heat capacity using the raw data as input. The raw data were not processed when the initial (*T*_1_) and final (*T*_2_) temperatures obtained during a heat capacity measurement straddled the saturation temperature. Each isochoric run was continued until the upper limit of either the temperature (300 K) or pressure (35 MPa) was obtained.

## 3. Results

**Figure 3:**
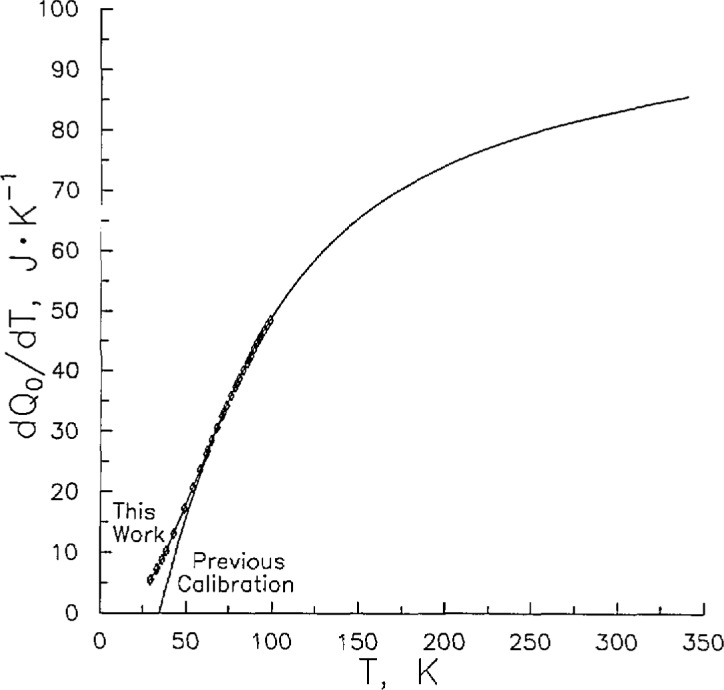
Heat capacity *C*_0_=d*Q*_0_/d*T* of the empty calorimeter: previous calibration [11]; this work (◊).

A significant adjustment must be applied to the raw heat capacity data for the energy required to heat the empty calorimeter from the initial (*T*_1_) to the final temperature (*T*_2_). For this work, *Q*_0_/*Q* ranged from 0.89 to 0.27. Since the published *Q*_0_ data had a lower limit of 86 K, experiments were conducted to extend the data to temperatures as low as 29 K. The results are shown in Table 1. An examination of the empty calorimeter’s heat capacity (*C*_0_) revealed that it is s-shaped when plotted against temperature. Further, *C*_0_ has a sharp curvature below 100 K. Combined, these properties make a high quality fit to raw *C*_0_ data difficult. In the face of these difficulties, efforts to define a *C*_0_(*T*) function were made by previous workers [6,9]. For this work, however, I fitted the data to the integral heat (*Q*_0_) function, Eq. (3), which is monotonic with no inflection. Values of Co can then be recovered from the derivative with temperature, *C*_0_ = d*Q*_0_/d*T*. Table 1 presents the raw data (*Q*_0_, *T*, Δ*T*), *Q*_0_ values calculated from the best fit to Eq. (3), and Co from an earlier study [11]. The coefficients of Eq. (3) are presented in Table 2. Calculated Co values establish that the new experimental measurements of *C*_0_ are both smooth and consistent with previous measurements to less than 0.19% at temperatures from 90 to 100 K, the region of overlap. This is depicted graphically in Fig. 3, which also shows that an extrapolation of our earlier calibration [11] would have led to serious errors at temperatures below 80 K.

The nitrogen sample used for this study is of very high purity. An analysis was furnished by the vendor. The impurities present in the research grade sample are 0.2 ppm CO_2_, 0.2 ppm total hydrocarbons, 1 ppm O_2_, and 1 ppm H_2_O. In addition, we performed our own analysis using gas chromatography-mass spectroscopy and confirmed these results.

The raw and reduced data for each run are presented in Table 3 for two-phase states, and in Table 4 for single-phase vapor and liquid states. Sufficient raw data are presented in Tables 3 and 4 to allow rechecking these computations or to reprocess the raw data using other equations for any adjustments to the experimental data. Data for the number of moles (*N*) in the calorimeter are provided in both Tables 3 and 4. These data identify and tie together the two-phase and single-phase portions of each isochoric run. Table 3 presents values of the two-phase heat capacity at constant volume (*C_v_*^(2)^) and the saturated liquid heat capacity (*C_σ_*) at the midpoint temperature (*T*) of each heating interval. Values of the saturated liquid heat capacity *C_σ_* are obtained by adjusting *C_v_*^(2)^ measurements with the thermodynamic relation,

$$\begin{array}{c} C_{\sigma }={C_{v}}^{\left(2\right)}-T\rho_{\sigma }^{-2}(d\rho_{\sigma }/dT)(dT_{\sigma }/dT)+\\ T[\rho_{\sigma }^{-1}-\rho^{-1}]d^{2}P_{\sigma }/dT^{2} \end{array}$$ (4)

where *ρ_σ_* and *P_σ_* are the density and pressure of the saturated liquid and *ρ* is the bulk density of the sample residing in the bomb. The derivative quantities were calculated using the formulation of Jacobsen et al. [8].

Corrections to the experimental heat capacity calculated using Eq. (2) for vaporization of sample are given by

$$C_{\Delta H}=\delta N_{c}\Delta H_{v}N^{-1}\Delta T^{-1}$$ (5)

where *δN*_c_ is the number of moles vaporized during a heating interval and Δ*H_v_* is the molar heat of vaporization calculated using the equation of state [8]. Thus, Eq. (5) corrects for the heat which drives a portion of the sample into the capillary by evaporation during a heat capacity experiment in the two-phase region. It is at most equal to 0.06% of *C_v_*^(2)^. In Table 3 the column labeled difference refers to calculations for *C_σ_* made with the equation of state in Ref. [8]. This equation of state correctly predicts the values within ±2%. Corrections for *PV* work on the bomb are given by

$$C_{PV}=k[T_{2}{\left(\partial P/\partial T\right)}_{\rho 2}-\Delta P/2]\Delta V_{m}/\Delta T$$ (6)

where *k* = 1000 J·MPa^−1^·dm^−3^, the pressure rise is Δ*P =P*_2_−*P*_1_, and the volume change per mole is $\Delta V_{m}=\rho_{2}^{-1}-\rho_{1}^{-1}$. The derivative has been calculated with the equation of state [8]. The *PV* work correction is important only for single-phase samples and varies between 0.26 and 3.8% of the value of *C_v_*. The largest such corrections occur for the highest density isochores.

**Figure 4:**
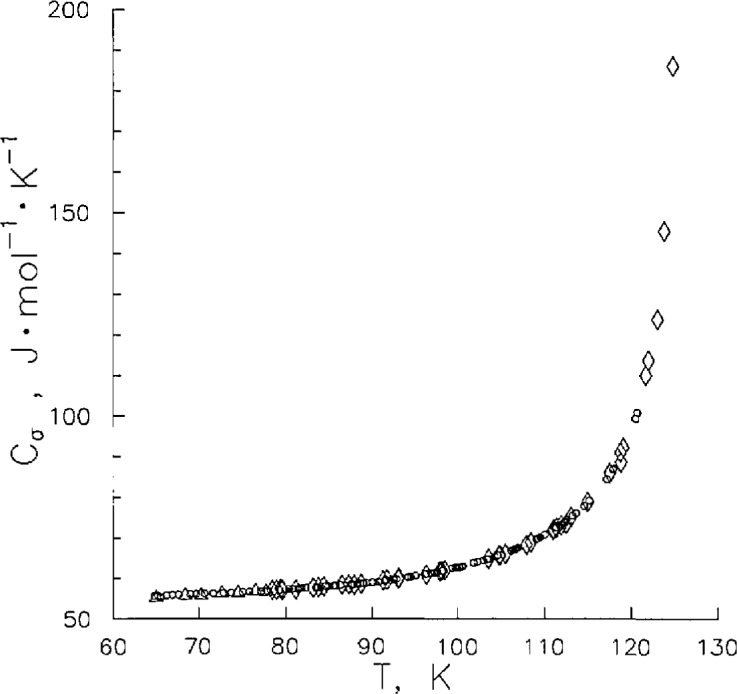
Saturated liquid heat capacity: Weber [2] (◊); Giauque and Clayton [3] (Δ); this work (○).

While we have observed that *C_v_*^(2)^ values are a function of both *T* and *ρ*, *C_σ_* values are a function of *T* only. Hence, *C_σ_* data provide us with a valuable check of the accuracy of our measurements by direct comparison with published data. Figure 4 shows the behavior of *C_σ_* from near the N_2_ triple point to near the critical point temperature where it rises sharply. Also shown in Fig. 4 are results of Weber [2] and Giauque and Clayton [3], whose data have uncertainties of ±0.5% and ±1%, respectively. In order to intercompare the data sets, our data were fitted to the expression,

$$C_{\sigma }=a+bT+cT^{2}+dT^{3}+eT/\sqrt{T-T_{c}.}$$ (7)

**Figure 5:**
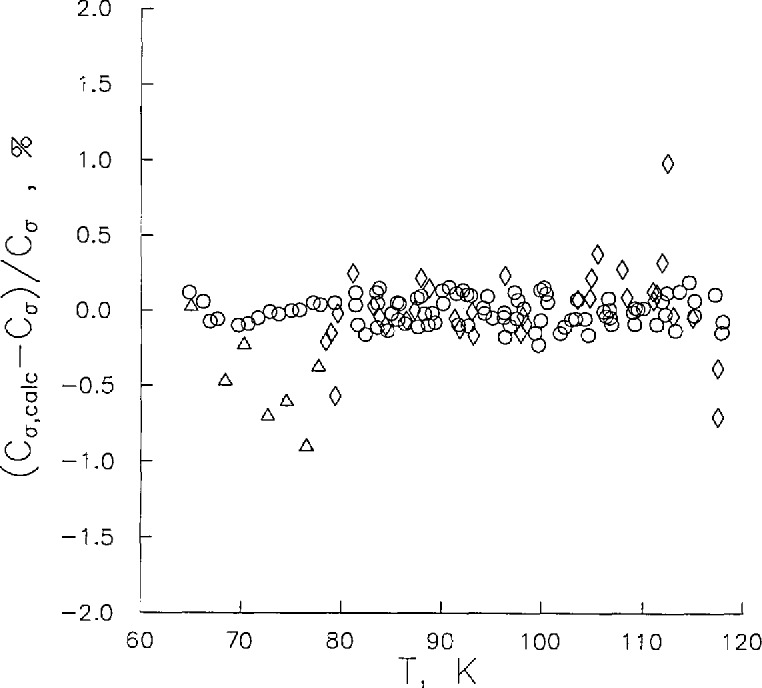
Deviations of saturated liquid heat capacity from Eq. (7): Weber [2] (◊); Giauque and Clayton [3] (Δ); this work (○) used In fit.

The coefficients of Eq. (7) are given in Table 5. Deviations of the *C_σ_* data of Refs. [2] and [3] from this expression were calculated also. The data of Refs. [2] and [3] were the most accurate available. This work overlaps the temperature range of both previous studies. The deviations of all the *C_σ_* measurements from Eq. (7) are shown in Fig. 5. We may conclude from Fig. 5 that the data of Refs. [2] and [3] are consistent with this work within ±1% with 95% of these data within ±0.2%.

**Figure 6:**
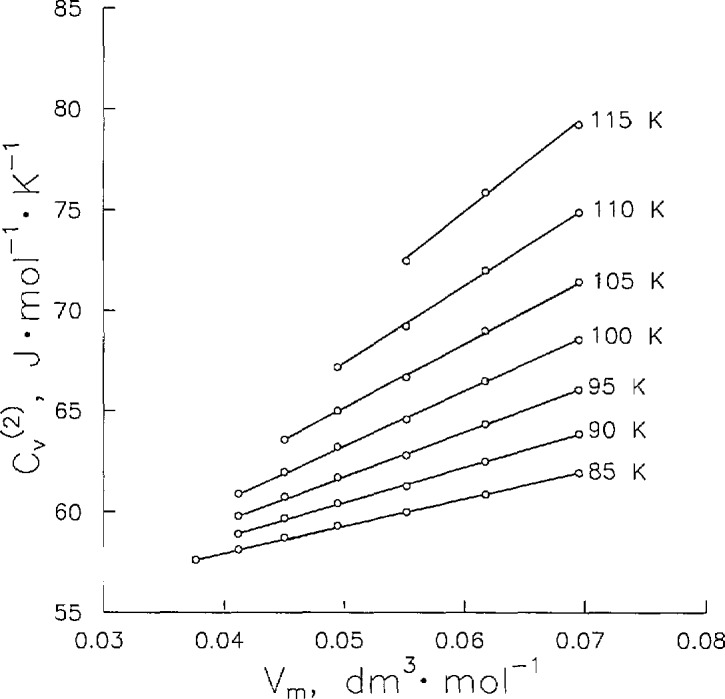
Two-phase heat capacity *C_v_*^(2)^ interpolated to integral temperatures.

It is also important to examine the internal consistency of our data. Perhaps the most interesting test of the internal consistency of the data derives from the relation

$${C_{v}}^{\left(2\right)}/T=-d^{2}\mu /dT^{2}+V_{m}d^{2}P_{\sigma }/dT^{2}$$ (8)

due to Yang and Yang [12], where *µ* is the chemical potential and *V*_m_ is the molar volume. This thermodynamic relation implies that when plotted on isotherms, *C_v_*^(2)^/*T* should be linear versus molar volume. To simplify this test, the measured *C_v_*^(2)^ data in Table 3 were fitted to the expression,

$${C_{v}}^{\left(2\right)}=a+bT+cT^{2}+dT/{\left(T-T_{c}\right)}^{0.1}$$ (9)

and new values were computed at integral temperatures from 65 to 125 K. Selected *C_v_*^(2)^ isotherms are shown in Fig. 6. We have observed that *C_v_*^(2)^ varies linearly with *V*_m_ within the experimental precision (±0.15%) of the data.

Further, we have obtained values of d^2^*P_σ_*/d*T*^2^ at integral temperatures, given in Table 6. Also shown in Table 6 are experimental values from Weber [2] and calculated values of this derivative which are from published vapor pressure equations [8,13]. The agreement of this work with published values is better than ±3 × 10^−5^ MPa · K^−2^.

**Figure 7:**
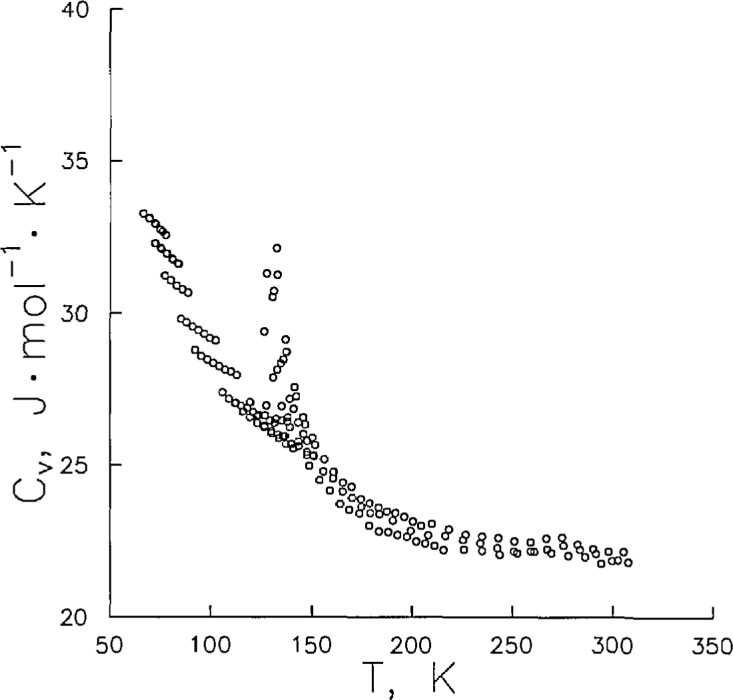
One-phase heat capacity *C_v_* at fourteen filling densities.

Values of the molar heat capacity at constant volume are depicted in Fig. 7. Shown in this plot are single-phase *C_v_* isochores at each of the 14 different filling densities of this work. As expected, *C_v_* increases with density up to the critical density (approx. 11.21 mol · dm^−3^), where it has a maximum value. Then at densities between the critical and twice the critical, *C_v_* decreases to a local minimum value. These data are found in Table 4. Also given in Table 4 is a column labeled “diff.” which gives the percent difference of this work from the equation of state in Ref. [8]. The authors of the equation of state estimate an accuracy of ± 2% for their calculated heat capacities. With only a few exceptions, these calculations are in fact within ±2% of the data. Most significantly, however, in the temperature range from 66 to 78 K, the values calculated with their equation fall 1 to 5% below the results of this study. Undoubtedly, accuracies would be improved by a new fit of the equation of state which includes this data.

**Figure 8:**
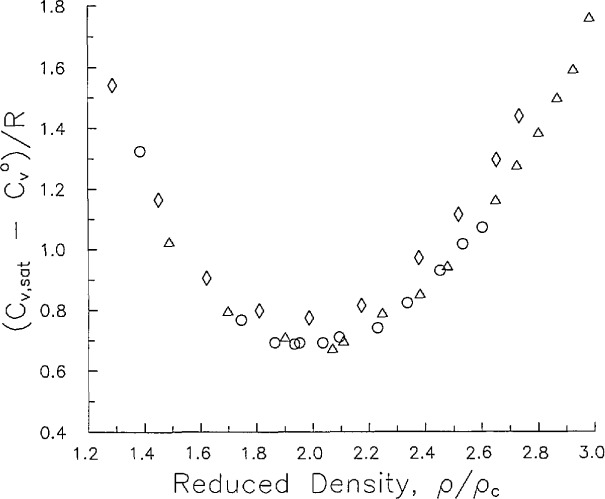
Reduced residual heat capacity evaluated at saturation plotted against reduced density; Ar (○) Ref. [14]; O_2_ (Δ) Ref. [14]; N_2_ (◊) this work.

At highly compressed liquid densities greater than twice critical, *C_v_* shows a rising trend which is indicative of hindered rotation of N_2_ molecules. A broad generalization can be made for low molecular mass gases with regard to the existence of a minimum liquid *C_v_* at 2.0 *ρ*_c_. If we examine a plot of reduced residual heat capacity $(C_{v}-C_{v}^{0})/R$ at saturated liquid states versus reduced density *ρ*/*ρ*_c_, shown in Fig. 8, we find identical behavior for Ar [14], O_2_ [14], and N_2_. A single parabola represents data for these three gases within experimental error. As shown by Fig. 8, the vertex of this parabola is found at 2.0 *ρ*_c_. I have not found a satisfactory explanation of this phenomenon based on firmly grounded theory. Further study is expected to lead to new insight and understanding of the behavior of liquid heat capacities.

## 4. Analysis of Errors

Uncertainty in *C_v_* arises from several sources. Primarily, the accuracy of this method is limited by how accurately we can measure the temperature rise. The platinum resistance thermometer has been calibrated on the IPTS-68 by NIST, with an uncertainty of ±0.002 K due to the calibration. Other factors, including gradients on the bomb, radiation to the exposed head of the thermometer, and time-dependendent drift of the ice point resistance lead to an overall uncertainty of *σ*_t_ = ± 0.03 K for the absolute temperature measurement. Uncertainty estimates of the relative temperature, however, are derived quite differently. The temperatures assigned to the beginning (*T*_1_) and to the end (*T*_2_) of a heating interval are determined by extrapolation of a linear drift (approximately −0.0005 K min^−1^) to the midpoint time of the interval. This procedure leads to an uncertainty of ±0.002 K for *T*_1_ and *T*_2_, and consequently ±0.004 K for the temperature rise, Δ*T = T*_2_*−T*_1_. For a typical experimental value of Δ*T* of 4 K, this corresponds to an uncertainty of ±0.1%. The energy applied to the calorimeter is the integral of the product of the applied potential and current from the initial to the final heating time; its uncertainty is ±0.01%. The energy applied to the empty calorimeter has been measured in repeated experiments and fitted to a function of temperature; the estimated uncertainty is ±0.02%. However, the adjustment is considerably larger for vapor than for liquid. For low density vapor the ratio *Q*_0_/*Q* is as large as 0.89, while for the highest density liquid it is as low as 0.27. This leads to considerably larger (approximately 10 times) uncertainty propagated to the heat capacity measurements for vapor states. The number of moles of each sample was determined within ± 0.2%. A correction for *PV* work on the bomb leads to an additional ±0.02% uncertainty. For pressures, the uncertainty due to the piston gauge calibration (± 0.05% max.) is added to the cross term [(*σ_t_*)(d*P*/d*T*)*_ρ_*] to yield an overall maximum probable uncertainty which varies from ±0.06 to ±0.8%, increasing steadily with the slope of the *P*(*ρ*, *T*) isochore to a maximum at the highest density and lowest pressure of the study. However, the pressure uncertainty does not appreciably contribute to the overall uncertainty for molar heat capacity. By combining the various sources of experimental uncertainty, I estimate the maximum uncertainty in *C_v_* which ranges from ±2.0% for vapor to ±0.5% for liquid.

## Figures and Tables

**Table 1:** Experimental heat values of the empty calorimeter

*T* K	Δ*T* K	*Q*_0_ J	*Q*_0,calc_ J	Diff.^b^ %	d*Q*_0_/d*T*^c^ J·K^−1^	*C*_0,calc_^d^ J·K^−1^	Diff.^e^ %
33.2131	12.0335	90.26328	90.49135	−0.3	7.501	−1.564	120.85
42.7043	6.9579	90.66839	90.76538	−0.1	13.031	8.785	32.58
48.8077	5.2636	90.47602	90.51839	−0.05	17.189	14.768	14.09
53.6257	4.3835	90.42722	90.35803	0.08	20.629	19.149	7.18
57.7245	3.8312	90.32820	90.20241	0.1	23.577	22.652	3.92
29.4295	4.0968	22.95437	22.89193	0.2	5.603	−6.072	208.37
33.0460	3.1274	22.88944	22.66654	1.0	7.319	−1.758	124.02
35.9052	2.5777	22.83842	22.59133	1.0	8.860	1.508	82.98
38.3089	2.2236	22.81414	22.57683	1.0	10.260	4.157	59.48
42.8741	6.9005	90.65877	90.78388	−0.1	13.138	8.958	31.81
48.9372	5.2363	90.48850	90.52425	−0.04	17.281	14.890	13.84
53.7321	4.3646	90.36904	90.30011	0.08	20.705	19.242	7.07
57.8148	3.8176	90.26715	90.12809	0.2	23.645	22727	3.88
61.4343	3.4396	90.19663	90.04630	0.2	26.223	25.655	2.17
64.7215	3.1554	90.04881	89.86412	0.2	28.538	28.189	1.22
67.7579	2.9392	89.98361	89.83021	0.2	30.615	30.427	0.61
70.5975	2.7668	89.96804	89.83038	0.2	32.517	32.436	0.25
73.2786	2.6229	89.88941	89.75887	0.1	34.271	34.260	0.03
75.8258	2.5022	89.86651	89.69035	0.2	35.915	35.930	−0.04
78.2613	2.3990	89.73939	89.61554	0.1	37.407	37.471	−0.17
80.5992	2.3119	89.73409	89.62376	0.1	38.814	38.901	−0.22
82.8526	2.2316	89.62329	89.46169	0.2	40.161	40.235	−0.18
85.0322	2.1666	89.61274	89.55121	0.07	41.361	41.485	−0.30
87.1452	2.1046	89.55704	89.45610	0.1	42.553	42.661	−0.25
89.1993	2.0501	89.55862	89.41201	0.2	43.685	43.770	−0.19
91.2015	1.9998	89.49705	89.31963	0.2	44.753	44.820	−0.15
93.1555	1.9544	89.42748	89.24169	0.2	45.757	45.817	−0.13
95.0632	1.9143	89.39781	89.22528	0.2	46.700	46.763	−0.14
96.9314	1.8760	89.35200	89.13514	0.2	47.629	47.666	−0.08
98.7621	1.8412	89.29452	89.06914	0.3	48.498	48.527	−0.06
62.3556	10.2210	274.4339	274.6478	−0.08	26.850	26.377	1.76
71.5780	8.2580	273.7197	273.9020	−0.07	33.146	33.111	0.11
79.2821	7.1899	273.2521	273.5237	−0.1	38.005	38.101	−0.25
86.1019	6.5020	272.9280	273.1325	−0.08	41.976	42.085	−0.26
92.3337	6.0173	272.6679	272.7770	−0.04	45.314	45.401	−0.19
29.1443	4.1877	22.95278	22.91044	0.2	5.481	−6.421	217.15
32.8341	3.1736	22.88800	22.66570	1.0	7.212	−2.005	127.80
35.7294	2.6077	22.85388	22.59940	1.0	8.764	1.311	85.05
38.1597	2.2428	22.80479	22.57232	1.0	10.168	3.996	60.70
42.7520	6.9371	90.60546	90.71014	−0.1	13.061	8.834	32.36
48.8448	5.2576	90.48330	90.55201	−0.08	17.210	14.803	13.99
53.6546	4.3758	90.36902	90.28945	0.09	20.652	19.174	7.16
57.7485	3.8264	90.26860	90.15482	0.1	23.591	22.672	3.90
61.3737	3.4432	90.16708	89.99313	0.2	26.187	25.607	2.21
64.6661	3.1618	90.09233	89.92512	0.2	28.494	28.147	1.22

a Equation (3).

b 100(*Q*_0_−*Q*_0_,_calc_)/*Q*_0_.

c Derived from Eq. (3).

d Reference [11].

e 100 (d*Q*_0_/d*T*−*C*_0,calc_/d*Q*_0_/d*T*.

**Table 2:** Coefficients of the function *Q*_0_(*T*), Eq. (3), of the empty calorimeter

*i*	*C*_*l*_
1	1.070179528057 · 10^1^
2	−4.721695058560 · 10^−1^
3	9.985458119236 · 10^−3^
4	3.443201289415 · 10^−6^
5	− 1.486069268038 · 10^−6^
6	1.901352098615 · 10^−8^
7	−1.300438485128 · 10^−10^
8	5.607423959480 · 10^−13^
9	− 1.572000054992 · 10^−15^
10	2.789945522377 · 10^−18^
11	−2.854347532609 · 10^−21^
12	1.284323931260 · 10^−24^

**Table 3:** Experimental two-phase heat capacities

*T* K	*ρ_σ_* mol·dm^−3^	*ρ_σ_* MPa	*N* mol	*V*_bomb_ cm^3^	Δ*T* K	*Q*/Δ*T* J·K^−1^	*C*_0_^a^ J·K^−1^	Adj.^b^ J·mol^−1^·K^−1^	Adj.^c^	*C_σ_* J·mol^−1^·K^−1^	*C_σ_*_,calc_^d^.	Diff.^e^ %	*C_v_*^(2)^ J·mol^−1^·K^−1^
67.634	30.358	0.027	2.1485	72.682	2.084	150.852	30.338	−0.001	0.013	55.99	56.68	−1.23	55.98
69.703	30.057	0.037	2.1485	7Z685	2.060	152.557	31.811	−0.001	0.038	56.16	57.08	−1.64	56.12
64.800	30.755	0.017	2.1485	72.678	2.120	148.342	28.248	−0.001	−0.007	55.71	55.66	0.09	55.72
66.904	30.462	0.024	2.1485	72.681	2.091	150.277	29.807	−0.001	0.007	55.95	56.48	−0.95	55.94
68.981	30.163	0.033	2.1485	72.683	2.067	152.020	31.302	−0.001	0.028	56.13	56.96	−1.49	56.10
65.223	30.697	0.018	2.0461	72.678	2.199	142.980	28.565	−0.001	−0.052	55.69	55.86	−0.30	55.75
71.685	29.760	0.050	2.0461	72.687	2.115	148.491	33.184	−0.001	−0.017	56.29	57.31	−1.81	56.30
73.785	29.436	0.066	2.0461	72.691	2.088	150.148	34.596	−0.002	0.016	56.46	57.45	−1.75	56.44
75.859	29.106	0.085	2.0461	72.694	2.066	151.757	35.951	−0.002	0.064	56.65	57.55	−1.59	56.58
77.912	28.772	0.109	2.0461	72.698	2.043	153.329	37.253	−0.002	0.129	56.86	57.64	−1.38	56.73
66.191	30.562	0.022	1.9346	72680	2.281	137.823	29.284	−0.001	−0.114	55.83	56.25	−0.75	55.95
70.686	29.911	0.043	1.9346	72686	2.216	141.662	32.497	−0.001	−0.127	56.23	57.21	−1.73	56.36
72.887	29.575	0.058	1.9346	72.689	2.189	143.357	33.998	−0.002	−0.119	56.37	57.40	−1.83	56.49
75.060	29.234	0.077	1.9346	72.693	2.161	145.116	35.434	−0.002	−0.099	56.57	57.51	−1.66	56.67
77.206	28.888	0.100	1.9346	72.6%	2.136	146.775	36.809	−0.002	−0.063	.77	57.61	−1.48	56.83
79.325	28.537	0.128	1.9346	72.700	2.111	148.482	38.127	−0.003	−0.008	57.03	57.72	−1.20	57.04
81.420	28.182	0.161	1.9346	72.704	2.086	150.150	39.392	−0.004	0.070	57.33	57.86	−0.94	57.2
83.492	27.822	0.199	1.9346	72.708	2.064	151.701	40.605	−0.004	0.175	57.61	58.06	−0.79	57.43
85.538	27.457	0.243	1.9346	72.712	2.040	153.400	41.770	−0.005	0.309	58.02	58.32	−0.52	57.71
87.564	27.087	0.293	1.9346	72.716	2.018	154.969	42.889	−0.006	0.479	58.42	58.65	−0.40	57.94
81.404	28.185	0.160	1.7683	72.704	2.210	141.220	39.382	−0.004	−0.314	57.28	57.86	−1.01	57.59
83.601	27.802	0.201	1.7683	72.708	2.181	143.094	40.669	−0.005	−0.257	57.67	58.07	−0.70	57.93
85.772	27.415	0.248	1.7683	72.712	2.152	144.875	41.901	−0.005	−0.169	58.07	58.36	−0.49	58.24
87.915	27.022	0.302	1.7683	72.717	2.126	146.576	43.080	−0.006	−0.043	58.49	58.72	−0.39	58.53
90.032	26.624	0.363	1.7683	72.721	2.101	148.271	44.210	−0.007	0.126	58.98	59.16	−0.31	58.85
92.122	26.220	0.433	1.7683	72.726	2.076	150.010	45.293	−0.008	0.345	59.56	59.69	−0.22	59.22
94.187	25.810	0.510	1.7683	72.731	2.048	151.854	46.332	−0.010	0.623	60.29	60.31	−0.03	59.67
96.227	25.392	0.595	1.7683	72.736	2.025	153.632	47.328	−0.011	0.970	61.08	61.03	0.08	60.11
98.246	24.965	0.690	1.7683	72.741	2.002	155.294	48.286	−0.012	1.396	61.90	61.84	0.09	60.50
100.239	24.530	0.793	1.7683	72.746	1.981	156.885	49.205	−0.013	1.915	62.79	6Z77	0.04	60.88
82.384	28.015	0.178	1.6152	72.706	2.645	134.159	39.961	−0.005	−0.742	57.59	57.95	−0.63	58.33
85.012	27.552	0.231	1.6152	72.711	2.604	136.253	41.474	−0.006	−0.728	57.96	58.25	−0.50	58.69
87.597	27.081	0.294	1.6152	72.716	2.560	138.526	42.908	−0.007	−0.668	58.54	58.66	−0.20	59.21
90.143	26.603	0.367	1.6152	72.722	2.522	140.545	44.269	−0.008	−0.551	59.06	59.19	−0.21	59.61
92.648	26.117	0.451	1.6152	72.727	2.480	142.835	45.561	−0.010	−0.364	59.86	59.84	0.03	60.22
95.116	25.621	0.548	1.6152	72.733	2.444	144.930	46.789	−0.011	−0.093	60.66	60.62	0.06	60.75
97.546	25.115	0.656	1.6152	72.739	2.407	147.066	47.957	−0.013	0.280	61.63	61.55	0.14	61.35
99.939	24.597	0.777	1.6152	72.745	2.371	149.220	49.069	−0.014	0.776	62.77	62.62	0.23	61.99
102.299	24.064	0.912	1.6152	72.751	2.336	151.445	50.128	−0.016	1.421	64.13	63.87	0.41	62.71
104.623	23.516	1.059	1.6151	72.758	2.300	153.724	51.137	−0.018	2.251	65.74	65.32	0.64	63.49
106.913	22.949	1.221	1.6151	72.764	2.267	155.933	52.099	−0.020	3.314	67.57	67.04	0.79	64.26
80.773	28.292	0.150	1.4726	72.703	2.828	125.246	39.005	−0.004	−1.186	57.38	57.81	−0.75	58.57
83.582	27.806	0.201	1.4725	72.708	2.776	127.586	40.658	−0.006	−1.281	57.76	58.07	−0.54	59.04
86.336	27.312	0.262	1.4725	72.714	2.722	129.958	42.215	−0.007	−1.336	58.26	58.44	−0.31	59.60
89.038	26.812	0.334	1.4725	72.719	2.674	132.254	43.684	−0.008	−1.337	58.82	58.94	−0.21	60.15
91.691	26.304	0.418	1.4725	72.725	2.624	134.661	45.073	−0.010	−1.272	59.57	59.57	−0.01	60.84
94.297	25.787	0.514	1.4725	72.731	2.579	136.915	46.386	−0.012	−1.126	60.35	60.35	0.00	61.47
96.859	25.260	0.624	1.4725	72.737	2.537	139.315	47.631	−0.013	−0.878	61.37	61.27	0.17	62.25
99.379	24.720	0.747	1.4725	72.744	2.494	141.680	48.812	−0.015	−0.507	62.54	62.35	0.31	63.05
101.858	24.165	0.885	1.4725	72.750	2.454	144.025	49.933	−0.017	0.015	63.89	63.62	0.43	63.88
104.296	23.595	1.038	1.4725	72.757	2.415	146.316	50.997	−0.020	0.728	65.43	65.10	0.51	64.71
106.695	23.004	1.205	1.4725	72.763	2.375	148.691	52.009	−0.022	1.683	67.31	66.86	0.67	65.63
109.051	22.391	1.388	1.4725	72.770	2.334	151.228	52.970	−0.024	2.952	69.65	68.98	0.96	66.70
111.367	21.749	1.585	1.4725	72.777	2.292	153.974	53.884	−0.027	4.646	72.58	71.61	1.35	67.94
113.643	21.069	1.799	1.4725	72.785	2.252	156.554	54.754	−0.029	6.947	76.04	74.98	1.40	69.10
81.647	28.143	0.165	1.3285	72.704	3.388	118.221	39.527	−0.005	−1.809	57.44	57.88	−0.78	59.25
84.998	27.554	0.230	1.3285	72.711	3.303	121.151	41.466	−0.007	−2.035	57.96	58.25	−0.49	59.99
88.268	26.957	0.312	1.3285	72.718	3.224	124.090	43.271	−0.009	−2.202	58.64	58.78	−0.24	60.84
91.461	26.349	0.410	1.3285	72.725	3.151	126.869	44.954	−0.011	−2.287	59.38	59.51	−0.23	61.66
94.579	25.730	0.525	1.3285	72.732	3.080	129.751	46.526	−0.013	−2.264	60.38	60.44	−0.10	62.64
97.629	25.097	0.660	1.3284	72.739	3.011	132.621	47.996	−0.015	−2.100	61.59	61.58	0.01	63.69
100.613	24.447	0.814	1.3284	72.747	2.945	135.501	49.375	−0.018	−1.757	63.05	62.95	0.16	64.81
103.532	23.776	0.988	1.3284	72.755	2.883	138.375	50.668	−0.021	−1.182	64.81	64.61	0.32	66.00
106.388	23.082	1.183	1.3284	72.763	2.819	141.416	51.882	−0.024	−0.300	67.07	66.62	0.67	67.37
109.182	22.356	1.398	1.3284	72.771	2.757	144.536	53.023	−0.027	1.001	69.85	69.11	1.06	68.85
111.918	21.589	1.635	1.3284	72.779	2.698	147.622	54.097	−0.030	2.900	73.26	72.34	1.27	70.36
114.590	20.769	1.894	1.3284	72.788	2.638	150.947	55.107	−0.034	5.719	77.82	76.71	1.43	72.10
117.198	19.872	2.174	1.3284	72.796	2.571	154.802	56.057	−0.037	10.092	84.38	83.09	1.54	74.29
83.765	27.773	0.204	1.1744	72.709	3.572	111.790	40.763	−0.007	−2.856	57.64	58.09	−0.79	60.49
90.722	26.492	0.385	1.1744	72.723	3.377	118.236	44.571	−0.012	−3.576	59.15	59.32	−0.29	62.73
97.307	25.165	0.645	1.1744	72.738	3.203	124.537	47.844	−0.017	−3.872	61.42	61.45	−0.05	65.29
100.473	24.478	0.806	1.1744	72.746	3.121	127.671	49.312	−0.020	−3.762	62.94	62.88	0.09	66.70
103.560	23.770	0.990	1.1744	72.755	3.045	130.854	50.680	−0.024	−3.405	64.83	64.62	0.32	68.24
106.571	23.036	1.196	1.1744	72.763	2.970	134.054	51.958	−0.027	−2.718	67.15	66.76	0.58	69.87
109.508	22.268	1.425	1.1744	72.772	2.897	137.424	53.153	−0.031	−1.569	70.15	69.45	1.00	71.72
112.372	21.456	1.677	1.1743	72.781	2.825	140.822	54.271	−0.035	0.256	73.91	72.98	1.27	73.65
115.163	20.582	1.953	1.1743	72.790	2.750	144.606	55.319	−0.039	3.157	79.14	77.88	1.59	75.98
117.873	19.619	2.251	1.1743	72.799	2.669	148.891	56.297	−0.043	7.971	86.77	85.29	1.70	78.80
120.500	18.507	2.572	1.1743	72.808	2.578	154.045	57.211	−0.047	16.851	99.26	98.10	1.17	82.41
81.860	28.106	0.168	1.0520	72.705	3.858	103.795	39.652	−0.007	−3.431	57.55	57.90	−0.60	60.99
85.660	27.435	0.245	1.0520	72.712	3.732	107.241	41.838	−0.009	−4.080	58.11	58.34	−0.40	62.19
89.337	26.756	0.342	1.0520	72.720	3.612	110.744	43.843	−0.012	−4.675	58.93	59.00	−0.13	63.60
92.902	26.066	0.461	1.0520	72.728	3.502	114.073	45.689	−0.015	−5.180	59.82	59.91	−0.15	65.00
96.354	25.365	0.601	1.0520	72.736	3.395	117.635	47.389	−0.018	−5.550	61.21	61.07	0.23	66.76
99.718	24.645	0.765	1.0520	72.744	3.303	121.054	48.967	−0.022	−5.741	62.76	62.51	0.40	68.50
102.980	23.906	0.953	1.0520	72.753	3.209	124.327	50.427	−0.026	−5.686	64.53	64.27	0.41	70.22
106.154	23.140	1.166	1.0519	72.762	3.122	127.718	51.784	−0.030	−5.298	66.85	66.43	0.62	72.14
109.240	22.340	1.403	1.0519	72.771	3.036	131.229	53.046	−0.034	−4.440	69.84	69.17	0.95	7428
112.240	21.495	1.665	1.0519	72.780	2.951	134.957	54.221	−0.039	−2.888	73.81	72.79	1.39	76.70
115.155	20.584	1.952	1.0519	72.789	2.865	138.905	55.316	−0.043	−0.211	79.20	77.87	1.68	79.41
117.981	19.577	2.263	1.0519	72.799	2.778	143.249	56.335	−0.048	4.516	87.08	85.68	1.62	82.57
120.707	18.408	2.598	1.0519	72.809	2.672	148.849	57.281	−0.052	13.769	100.76	99.56	1.19	86.99
80.375	28.360	0.144	0.8789	72.702	4.307	93.168	38.766	−0.007	−4.560	57.35	57.78	−0.76	61.91
84.593	27.626	0.222	0.8789	72.710	4.121	97.154	41.237	−0.010	−5.688	57.95	58.19	−0.42	63.64
88.638	26.887	0.322	0.8789	72.718	3.954	101.081	43.470	−0.013	−6.791	58.77	58.86	−0.15	65.56
92.528	26.140	0.447	0.8789	72.727	3.813	104.844	45.500	−0.017	−7.818	59.70	59.80	−0.17	67.52
96.277	25.381	0.598	0.8789	72.736	3.674	108.706	47.352	−0.022	−8.715	61.08	61.04	0.06	69.79
99.896	24.606	0.775	0.8789	72.745	3.550	112.394	49.049	−0.026	−9.422	62.62	62.60	0.04	72.04
103.393	23.809	0.979	0.8789	72.754	3.430	116.261	50.607	−0.031	−9.860	64.80	64.52	0.43	74.66
106.778	22.983	1.211	0.8789	72.764	3.319	120.056	52.043	−0.037	−9.921	67.42	66.93	0.72	77.34
110.053	22.119	1.471	0.8789	72.773	3.214	123.926	53.369	−0.042	−9.432	70.79	70.04	1.07	80.22
113.221	21.200	1.758	0.8788	72.783	3.108	128.099	54.594	−0.048	−8.089	75.49	74.27	1.60	83.57

a Derived from Eq. (3). *C*_0_=d*Q*_0_/d*T.*

b Equations (5) and (6).

c Equation (4).

d Reference [8].

e 100(*C_σ_*−*C_σ_*_,calc_)/*C_σ_*.

**Table 4:** Experimental single-phase heat capacities

*T* K	*ρ* mol·dm^−3^	*P* MPa	*N* mol	*V*_bomb_ cm^3^	Δ*T* K	*Q*/Δ*T* J·K^−1^	*C*_0_^a^ J·K^−1^	Adj.^b^	*C_v_* J·mol^−1^·K^−1^	*C_v_*_,calc_^c^	Diff.^d^ %
66.1756	30.9599	8.5838	2.2520	72.739	2.9305	107.357	29.273	1.28	33.26	31.76	4.526
9.0867	30.8819	14.4587	2.2477	72.785	2.8898	108.710	31.377	1.24	33.09	31.98	3.33
71.9548	30.8328	20.2568	*22456*	72.832	2.8506	110.186	33368	1.22	32.95	32.13	2.49
74.7849	30.8035	25.9553	2.2449	72.879	2.8157	111.573	35.254	1.21	32.76	32.18	1.76
77.5788	30.7869	31.5376	2.2452	72.927	2.7814	112.954	37.044	1.22	3238	32.17	1.25
69.2774	30.8813	14.9188	2.2478	72.789	3.4450	108.863	31.512	1.23	33.11	32.00	3.34
72.6946	30.8246	21.7709	2.2454	72.844	3.3915	110.553	33.868	1.21	32.91	32.15	2.30
76.0585	30.7943	28.4964	2.2449	72.901	3.3428	112.208	36.079	1.21	32.69	32.19	1.53
72.6097	30.3311	11.8026	2.2073	72.773	2.9139	107.734	33.811	1.16	32.29	31.30	3.06
75.5013	30.2825	17.2998	2.2051	72.818	2.8762	109.104	35.720	1.16	32.11	31.41	2.17
78.3559	30.2515	22.6862	2.2042	72.864	2.8386	110.564	37.530	1.16	31.97	31.46	1.61
81.1743	30.2318	27.9586	2.2042	72.910	2.8076	111.831	39.245	1.18	31.76	31.45	0.99
83.9583	30.2188	33.1070	2.2046	72.956	2.7718	113.210	40.874	1.20	31.62	31.39	0.73
72.0307	30.3444	10.7150	2.2080	72.764	2.9234	107.377	33.419	1.17	32.29	31.27	3.14
74.9320	30.2907	16.2231	2.2054	72.809	2.8824	108.874	35.350	1.16	32.16	31.40	2.38
77.7934	30.2568	21.6323	2.2044	72.855	2.8466	110.217	37.179	1.16	31.97	31.45	1.62
80.6178	30.2352	26.9234	2.2042	72.901	2.8122	111.579	38.912	1.17	31.80	31.45	1.10
83.4075	30.2209	32.0913	2.2045	72.947	2.7793	112.913	40.557	1.19	31.64	31.41	0.73
77.0893	29.5526	8.6948	2.1502	72.759	2.9544	106.205	36.736	1.06	31.24	30.51	2.34
80.0185	29.5055	13.7919	2.1481	72.802	2.9149	107.584	38.550	1.07	31.08	30.57	1.62
82.9065	29.4743	18.7781	2.1471	72.846	2.8759	108.936	40.266	1.08	30.91	30.60	1.02
85.7558	29.4528	23.6465	2.1468	72.889	2.8404	110.320	41.892	1.10	30.79	30.58	0.67
88.5715	29.4368	28.3997	2.1469	72.933	2.8069	111.676	43.434	1.12	30.68	30.53	0.46
85.2388	28.1342	6.0948	2.0469	72.756	2.9975	104.445	41.602	0.89	29.82	29.39	1.46
88.2096	28.0972	10.4732	2.0453	72.795	2.9579	105.825	43.239	0.91	29.70	29.37	1.12
91.1399	28.0717	14.7565	2.0446	72.835	2.9212	107.095	44.788	0.93	29.56	29.35	0.70
94.0353	28.0522	18.9399	2.0443	72.874	2.8869	108.360	46.257	0.95	29.44	29.31	0.41
96.8947	28.0364	23.0260	2.0442	72.914	2.8548	109.573	47.648	0.97	29.32	29.26	0.20
99.7227	28.0232	27.0294	2.0444	72.953	2.8259	110.653	48.970	1.00	29.18	29.20	−0.07
102.5197	28.0104	30.9381	2.0446	72.993	2.7951	111.823	50.225	1.02	29.10	29.12	−0.05
92.3233	26.5920	3.1853	1.9345	72.748	3.0508	102.476	45.396	0.73	28.79	28.46	1.13
95.3475	26.5632	6.9012	1.9334	72.783	3.0172	103.613	46.902	0.75	28.59	28.35	0.86
98.3344	26.5439	10.5495	1.9329	72.819	2.9797	104.856	48.328	0.77	28.48	28.28	0.70
101.2850	26.5288	14.1182	1.9327	72.855	2.9451	106.022	49.677	0.79	28.36	28.23	0.48
104.2006	26.5166	17.6147	1.9328	72.890	2.9134	107.127	50.956	0.81	28.25	28.17	0.27
107.0820	26.5049	21.0309	1.9329	72.926	2.8842	108.191	52.169	0.83	28.14	28.11	0.13
109.9340	26.4947	24.3895	1.9331	72.961	2.8541	109.272	53.322	0.86	28.08	28.04	0.14
1127578	26.4840	27.6783	1.9333	72.997	2.8304	110.181	54.419	0.88	27.96	27.97	−0.04
105.7600	24.2453	4.8752	1.7648	72.791	3.0766	100.935	51.619	0.55	27.39	27.28	0.40
108.8264	24.2371	7.7000	1.7650	72.822	3.0460	101.875	52580	0.57	27.18	27.11	0.26
111.8576	24.2317	10.4814	1.7653	72.852	3.0164	102.853	54.074	0.59	27.03	27.00	0.11
114.8599	24.2259	13.2159	1.7657	72.883	2.9831	103.887	55.207	0.61	26.95	26.92	0.12
117.8323	24.2205	15.9078	1.7660	72.913	2.9560	104.861	56.283	0.63	26.87	26.85	0.08
120.7784	24.2140	18.5546	1.7663	72.944	2.9311	105.701	57.306	0.65	26.74	26.78	−0.13
123.6977	24.2071	21.1602	1.7665	72.975	2.9064	106.567	58.279	0.67	26.66	26.71	−0.18
126.5950	24.1991	23.7239	1.7667	73.005	2.8807	107.485	59.206	0.69	26.64	26.64	−0.03
129.4678	24.1907	26.2495	1.7668	73.036	2.8633	108.096	60.090	0.70	26.47	26.58	−0.43
132.3201	24.1817	28.7374	1.7669	73.067	2.8364	109.073	60.934	0.72	26.52	26.51	0.04
135.1429	24.1724	31.1832	1.7669	73.098	2.8135	109.817	61.738	0.74	26.47	26.45	0.09
137.9433	24.1626	33.5919	1.7670	73.128	2.7945	110.542	62.506	0.75	26.43	26.39	0.17
112.3409	22.1595	3.1882	1.6131	72.793	3.5832	98.547	54.260	0.41	27.04	27.02	0.07
115.9124	22.1468	5.6836	1.6128	72.823	3.5489	99.454	55.593	0.43	26.76	26.59	0.64
119.4468	22.1408	8.1621	1.6130	72.852	3.5135	100.436	56.849	0.45	26.57	26.35	0.85
122.9472	22.1381	10.6204	1.6135	72.883	3.4780	101.363	58.032	0.46	2639	26.19	0.76
126.4129	22.1302	13.0294	1.6136	72.913	3.4438	102.298	59.149	0.48	26.25	26.07	0.71
129.8462	22.1242	15.4143	1.6138	72.943	3.4128	103.165	60.204	0.50	26.12	25.97	0.57
133.2459	22.1177	17.7654	1.6140	72.974	3.3829	104.003	61.201	0.52	26.00	25.88	0.46
136.6150	22.1102	20.0821	1.6141	73.004	3.3532	104.893	62.145	0.54	25.95	25.80	0.58
119.3182	20.2246	3.7560	1.4727	72.815	3.6281	97.117	56.804	0.31	27.06	27.03	0.08
122.9365	20.2050	5.7012	1.4717	72.841	3.6044	97.697	58.029	0.33	26.62	26.41	0.80
126.5253	20.1948	7.6456	1.4715	72.867	3.5761	98.407	59.185	0.35	26.30	26.04	1.01
130.0849	20.1876	9.5802	1.4715	72.893	3.5480	99.139	60.276	0.36	26.05	25.80	0.94
133.6139	20.1808	11.4985	1.4716	72.920	3.5169	99.966	61.307	0.38	25.89	25.63	1.01
137.1129	20.1750	13.4013	1.4717	72.947	3.4894	100.687	62.281	0.39	25.70	25.50	0.81
140.5803	20.1679	15.2807	1.4717	72.974	3.4628	101.401	63.203	0.41	25.55	25.38	0.65
147.4210	20.1529	18.9728	1.4717	73.028	3.4039	102.823	64.903	0.44	25.33	25.19	0.57
150.8021	20.1443	20.7859	1.4716	73.055	3.3782	103.547	65.689	0.45	25.28	25.10	0.69
127.3163	18.2448	5.2548	1.3291	72.848	4.1564	95.609	59.432	0.25	26.97	26.86	0.41
131.4649	18.2238	6.9785	1.3280	72.874	4.1314	96.084	60.685	0.27	26.39	26.15	0.90
135.5815	18.2155	8.7087	1.3279	72.901	4.1031	96.688	61.860	0.28	25.95	25.73	0.84
139.6662	18.2082	10.4320	1.3279	72.928	4.0666	97.462	62.964	0.30	25.69	25.45	0.93
126.4530	16.2966	3.8154	1.1869	72.834	4.2098	94.283	59.162	0.17	29.41	29.79	−1.26
130.6765	16.0985	5.0768	1.1729	72.856	4.2449	93.387	60.452	0.19	27.89	27.83	0.21
134.9085	16.0823	6.4036	1.1721	72879	4.2317	93.468	61.672	0.20	26.93	26.67	0.96
139.1213	16.0846	7.7470	1.1726	72.902	4.2110	93.845	62.820	0.22	26.25	25.99	0.99
143.3060	16.0869	9.0928	1.1732	72926	4.1806	94.410	63.898	0.23	25.78	25.55	0.92
147.4574	16.0876	10.4346	1.1736	72951	4.1517	95.028	64.911	0.24	25.43	25.24	0.74
130.2059	14.1938	4.4025	1.0340	72849	4.3099	92.049	60.312	0.15	30.54	30.34	0.67
134.5362	14.2878	5.4914	1.0412	72.870	4.3446	91.257	61.568	0.16	28.36	27.98	1.33
138.8786	14.3485	6.6081	1.0459	72.892	4.3393	91.358	62.756	0.17	27.18	26.72	1.70
143.2083	14.3826	7.7332	1.0487	72914	4.3162	91.743	63.874	0.18	26.40	25.98	1.59
147.5123	14.4028	8.8591	1.0505	72.936	4.2922	92214	64.924	0.19	25.79	25.49	1.14
132.0747	11.7620	4.4567	0.8569	72.854	4.4820	88.512	60.863	0.12	32.14	31.55	1.86
136.5879	11.9194	5.3117	0.8686	72.874	4.5294	87.555	62.138	0.13	29.13	28.63	1.75
141.1204	11.9827	6.1771	0.8735	72.894	4.5279	87.530	63.343	0.14	27.56	27.08	1.74
145.6394	12.0146	7.0439	0.8760	72915	4.5080	87.872	64.474	0.15	26.57	26.17	1.49
150.1322	12.0316	7.9074	0.8775	72.935	4.4773	88.395	65.535	0.15	25.90	25.58	1.24
132.5996	10.2076	4.3573	0.7437	72.855	4.7041	84.345	61.015	0.11	31.27	31.44	−0.55
137.3223	10.1724	5.0568	0.7413	72.874	4.7362	83.715	62.338	0.11	28.73	28.59	0.47
142.0505	10.1587	5.7565	0.7405	72.893	4.7206	83.850	63.581	0.12	27.26	27.05	0.79
146.7525	10.1507	6.4513	0.7401	72912	4.6912	84.319	64.743	0.12	26.33	26.12	0.82
151.4228	10.1446	7.1397	0.7399	72.932	4.6574	84.904	65.829	0.13	25.66	25.50	0.63
156.0526	10.1406	7.8212	0.7398	72.952	4.6183	85.575	66.844	0.13	25.19	25.04	0.57
160.6436	10.1369	8.4954	0.7397	72972	4.5785	86.222	67.795	0.14	24.77	24.69	0.34
165.1919	10.1336	9.1617	0.7397	72992	4.5417	86.855	68.686	0.14	24.42	24.40	0.08
169.6996	10.1302	9.8204	0.7396	73.012	4.5066	87.610	69.523	0.15	24.30	24.15	0.61
174.1710	10.1264	10.4716	0.7395	73.032	4.4733	88.099	70.311	0.15	23.89	23.94	−0.21
178.6041	10.1225	11.1153	0.7395	73.052	4.4369	88.735	71.055	0.16	23.75	23.76	−0.05
183.0001	10.1189	11.7521	0.7394	73.072	4.4042	89.342	71.757	0.16	23.61	23.60	0.08
187.3635	10.1153	12.3825	0.7393	73.092	4.3729	89.907	72.423	0.17	23.48	23.45	0.10
191.6890	10.1125	13.0069	0.7393	73.112	4.3382	90.510	73.054	0.17	23.43	23.33	0.47
195.9802	10.1105	13.6259	0.7394	73.132	4.3119	91.024	73.653	0.18	23.31	23.21	0.44
200.2466	10.1079	14.2391	0.7394	73.152	4.2886	91.478	74.225	0.18	23.15	23.11	0.18
204.4752	10.1060	14.8466	0.7395	73.172	4.2636	91.925	74.769	0.19	23.01	23.02	−0.02
210.0585	10.1008	15.6420	0.7394	73.198	8.4399	92.646	75.456	0.20	23.06	22.91	0.65
218.3924	10.0959	16.8298	0.7394	73.239	8.3530	93.507	76.421	0.20	22.90	22.77	0.58
226.6318	10.0911	17.9990	0.7395	73.279	8.2747	94.275	77.311	0.21	22.72	2265	0.29
234.7910	10.0834	19.1453	0.7393	73.319	8.1942	95.062	78.136	0.22	22.66	22.55	0.47
242.8663	10.0801	20.2847	0.7395	73.359	8.1251	95.797	78.902	0.23	22.60	2247	0.60
250.8702	10.0748	21.4044	0.7395	73.399	8.0608	96.445	79.617	0.24	22.51	22.40	0.53
258.8035	10.0694	22.5096	0.7395	73.439	7.9993	97.065	80.285	0.25	22.46	22.33	0.56
266.6768	10.0636	23.6003	0.7395	73.479	7.9311	97.777	80.912	0.26	22.60	22.28	1.40
274.4971	10.0578	24.6790	0.7394	73.519	7.8800	98.376	81.500	0.27	22.63	22.24	1.74
2822634	10.0524	25.7471	0.7394	73.558	7.8414	98.751	82.054	0.27	22.40	2220	0.91
289.9925	10.0466	26.8042	0.7394	73.598	7.7978	99.164	82.577	0.28	22.25	22.16	0.39
297.6971	10.0405	27.8527	0.7394	73.638	7.7563	99.611	83.075	0.29	22.16	22.13	0.12
305.4009	10.0343	28.8964	0.7393	73.679	7.7187	100.111	83.551	0.30	22.15	22.11	0.21
130.8320	8.3415	3.9188	0.6076	72.846	5.0045	79.223	60.498	0.09	30.73	31.55	−2.69
135.8440	8.2185	4.4754	0.5988	72864	5.0201	79.040	61.933	0.09	28.48	28.47	0.04
140.8426	8.1615	5.0222	0.5948	72883	4.9925	79.288	63.271	0.10	26.84	26.86	−0.08
145.7985	8.1292	5.5589	0.5926	72.902	4.9437	79.989	64.513	0.10	26.02	25.90	0.46
150.7101	8.1089	6.0866	0.5913	72.920	4.8982	80.696	65.668	0.11	25.32	25.26	0.23
155.5657	8.0962	6.6057	0.5905	72.939	4.8486	81.440	66.740	0.11	24.79	24.79	−0.01
160.3678	8.0871	7.1162	0.5900	72.958	4.7965	82.299	67.739	0.11	24.56	24.42	0.57
165.1232	8.0808	7.6198	0.5897	72.977	4.7509	82.975	68.673	0.12	24.13	24.12	0.04
169.8250	8.0756	8.1153	0.5895	72.996	4.7024	83.729	69.546	0.12	23.93	23.87	0.26
174.4834	8.0714	8.6044	0.5893	73.015	4.6666	84.377	70.365	0.13	23.64	23.66	−0.05
179.1053	8.0673	9.0873	0.5892	73.034	4.6259	85.022	71.136	0.13	23.43	23.47	−0.16
183.6860	8.0631	9.5638	0.5890	73.053	4.5857	85.724	71.864	0.14	23.39	23.31	0.36
190.3080	8.0587	10.2509	0.5889	73.080	9.0408	86.587	72855	0.14	23.18	23.11	0.32
199.2457	8.0544	11.1748	0.5889	73.118	8.9245	87.626	74.093	0.15	22.84	2288	−0.19
208.0556	8.0502	120803	0.5889	73.156	8.8087	88.670	75.214	0.16	22.70	22.70	−0.01
216.7534	8.0455	12.9687	0.5889	73.193	8.6985	89.692	76.237	0.16	22.68	22.55	0.58
225.3505	8.0409	13.8425	0.5888	73.231	8.6044	90.566	77.177	0.17	22.55	22.43	0.56
233.8541	8.0362	14.7024	0.5888	73.269	8.5230	91.372	78.044	0.18	2244	2232	0.51
242.2773	8.0314	15.5501	0.5888	73.306	8.4479	92085	78.848	0.19	2228	22.24	0.18
250.6135	8.0268	16.3857	0.5887	73.344	8.3741	92.764	79.595	0.19	2217	2217	0.03
258.8895	8.0217	17.2106	0.5886	73.381	8.3097	93.448	80.292	0.20	22.17	22.10	0.31
267.1020	8.0167	18.0258	0.5886	73.419	8.2442	94.122	80.944	0.21	22.24	22.05	0.86
275.2560	8.0116	18.8314	0.5885	73.456	8.1783	94.784	81.556	0.22	22.36	22.00	1.59
283.3544	8.0065	19.6284	0.5884	73.493	8.1280	95.267	82129	0.22	22.22	21.96	1.18
291.4014	8.0018	20.4184	0.5884	73.531	8.0823	95.733	82.670	0.23	2209	21.93	0.75
299.4287	7.9968	21.2024	0.5883	73.568	8.0399	96.127	83.183	0.24	21.86	21.90	−0.16
307.4412	7.9916	21.9817	0.5882	73.606	7.9875	96.620	83.674	0.24	21.82	21.87	−0.25
127.3144	6.0901	3.2938	0.4435	72.831	5.4232	73.354	59.431	0.08	31.31	30.48	2.66
132.7254	6.1679	3.7382	0.4493	72.850	5.3865	73.729	61.052	0.08	28.14	27.70	1.56
138.0861	6.1711	4.1600	0.4497	72.868	5.3243	74.524	62544	0.08	26.57	26.21	1.37
143.3730	6.1676	4.5679	0.4495	72.886	5.2530	75.465	63.915	0.08	25.62	25.32	1.18
148.5902	6.1628	4.9645	0.4493	72904	5.1815	76.438	65.178	0.09	24.98	24.73	1.02
153.7331	6.1594	5.3516	0.4492	72.922	5.1152	77.392	66.343	0.09	24.51	24.29	0.90
158.8084	6.1569	5.7305	0.4491	72.941	5.0505	78.312	67.421	0.10	24.16	23.95	0.84
163.8194	6.1554	6.1022	0.4491	72.959	4.9947	79.120	68.422	0.10	23.72	23.68	0.19
168.7682	6.1542	6.4671	0.4491	72.977	4.9387	79.975	69.354	0.10	23.54	23.45	0.41
173.6621	6.1533	6.8261	0.4492	72.995	4.8845	80.791	70.223	0.11	23.42	23.25	0.71
178.5030	6.1525	7.1796	0.4492	73.013	4.8418	81.426	71.038	0.11	23.01	23.08	−0.31
183.2876	6.1519	7.5277	0.4493	73.031	4.7966	82.111	71.802	0.11	22.83	2293	−0.46
188.0301	6.1506	7.8706	0.4493	73.050	4.7514	82.818	72.522	0.12	2280	2281	−0.03
1927222	6.1498	8.2091	0.4494	73.068	4.7141	83.461	73.200	0.12	22.71	2269	0.10
197.3785	6.1487	8.5435	0.4494	73.086	4.6762	84.077	73.843	0.12	22.65	22.59	0.27
201.9963	6.1476	8.8742	0.4494	73.104	4.6439	84.619	74.453	0.13	22.50	22.50	−0.02
206.5742	6.1467	9.2013	0.4495	73.122	4.6119	85.171	75.032	0.13	22.43	2242	0.04
211.1170	6.1455	9.5246	0.4495	73.140	4.5784	85.692	75.583	0.13	2235	22.35	0.03
215.6256	6.1443	9.8447	0.4495	73.157	4.5517	86.159	76.109	0.14	2222	22.28	−0.29
225.9475	6.1421	10.5756	0.4496	73.199	8.9395	87.304	77.239	0.14	2223	2215	0.34
234.7724	6.1397	11.1968	0.4496	73.235	8.8400	88.190	78.134	0.15	22.19	2206	0.60
243.4888	6.1378	11.8087	0.4497	73.270	8.7463	88.958	78.959	0.16	22.06	21.98	0.34
252.1201	6.1352	12.4110	0.4497	73.306	8.6624	89.740	79.725	0.16	22.11	21.92	0.87
260.6670	6.1324	13.0049	0.4498	73.341	8.5795	90.456	80.437	0.17	2215	21.86	1.32
269.1402	6.1297	13.5919	0.4498	73.377	8.5140	91.088	81.100	0.18	2212	21.81	1.41
277.5386	6.1271	14.1718	0.4498	73.412	8.4513	91.647	81.720	0.18	22.02	21.77	1.16
285.8948	6.1237	14.7451	0.4498	73.447	8.3925	92.204	82.303	0.19	21.99	21.73	1.16
294.1859	6.1206	15.3127	0.4498	73.483	8.3413	92.662	82.851	0.19	21.77	21.70	0.32
302.4355	6.1171	15.8745	0.4497	73.518	8.2861	93.249	83.370	0.20	21.88	21.68	0.92

a Derived from Eq. (3). *C*_0_=d*Q*_0_/d*T.*

b Equation (6).

c Reference [8].

d 100(*C*_0_−C*_v_*_,calcc_)/*C_v_*.

**Table 5:** Coefficients of the function *C_σ_*(*T*), Eq. (7)^a^,^b^

Coefficient	Value
*a*	0.469355 × 10^2^
*b*	0.211629×10^0^
*c*	−0.490463 ×10^−4^
*d*	0.184354 ×10^−4^
*e*	0.129524 ×10^−1^

a Units are J · mol^−1^ · K^−1^ and K.

b Temperature range is 64 to 118 K.

**Table 6:** Comparison of the vapor pressure second derivatives d^2^*P*/d*T*^2^ from heat capacity measurements with published experimental values and with values from vapor pressures

*T*,K	(d^2^*P*/d*T*^2^) ×10^5^ MPa·K^−2^			
	Experimental		Calculated	
	This work	Weber [2]	Jacobsen et al. [8]	Goodwin [13]
85	157		158	158
90	192	195	194	194
95	232		231	232
100	270	270	270	271
105	308		310	312
110	353	355	354	355
115	409		407	403

## 5. Appendix 1. Calorimeter Volumes

A knowledge of the volumes of connecting tubing, couplings, valves, and so on is a valuable aid when deducing certain adjustments to raw measurements. The bomb volume is the same as reported in detail by Goodwin and Weber [9] and is given as a function of temperature and pressure by

$$V_{\text{bomb}}=V_{r}(1.0+3.0(C_{1}+C_{2}T_{r})e^{\alpha (1-1/T_{r}\text{)}}+k{T_{r}}^{1/3}P)$$ (10)

where*V*_r_=72.657 cm^3^, *T*_r_=T/100, *C*_1_= −2.1461 ×10^−4^, *C*_2_ = 5.9455×10^−4^, *α*= 1.01062, and *k* = 1.09548 × 10^−4^ MPa^−1^. The appropriate units to be used with Eq. (10) are temperature in K and pressure in MPa.

For this apparatus, all elements of volume except for the bomb are called noxious volumes. Extensive changes were made to the noxious volumes prior to this work. In Fig. 1, the bomb is connected to the pressure transducer with a 71 cm length of fine bore (ID = 0.015 cm) capillary tubing, which passes vertically from the cryostat. Of this length, 62.5 cm is outside the adiabatic zone of the calorimeter. This volume (0.01295 cm^3^) is combined with contributions from a three-port valve (0.008 cm^3^), an additional 8.79 cm length of medium bore (ID = 0.051 cm) capillary (0.0178 cm^3^), and the pressure transducer (0.0745 cm^3^) for a total noxious volume of 0.1132 cm^3^. In the worst case, the total noxious volume is only about 0.15% of the bomb volume. These last three volumes are thermostatted in an aluminum block oven, and maintained by a proportional controller at 320.0 ± 0.05 K. This oven serves two purposes. It provides a stable environment for the internal electronics of the pressure transducer and it fixes the temperature of the upper end of the 71 cm long capillary at a temperature sufficient to drive the vapor-liquid meniscus down to near the guard ring. Since only vapor resides in most of the noxious volumes, the ratio of the number of moles in these volumes to those in the bomb ranges from 1 part in 10^4^ for vapor to 1 part in 10^6^ for liquid.

## Glossary

*a,b,c,d,e*: Coefficients for Eq. (7)
*C_v_*: Molar heat capacity at constant volume
$C_{v}^{0}$,: Molar heat capacity in the ideal gas state
*C_v_*^(2)^: Molar heat capacity of a two-phase sample
*C_σ_*: Molar heat capacity of a saturated liquid sample
*V*_bomb_: Volume of the calorimeter containing sample
*V*_m_: Molar volume, dm^3^ · mol −^1^
*P*: Pressure, MPa
*P_σ_*: Vapor pressure
Δ*P*: Pressure rise during a heating interval
*T*: Temperature, K
*T*_c_: Critical-point temperature
*T*_1_*T*_2_: Temperature at start and end of heating interval
Δ*T*: Temperature rise during a heating interval
*Q*: Calorimetric heat energy input to bomb and sample
*Q*_0_: Calorimetric heat energy input to empty bomb
*N*: Moles of substance in the calorimeter
*ρ*: Fluid density, mol·dm−^3^
*ρ_σ_*: Saturated liquid density
*µ*: Chemical potential
